# Crab Spider Lures Prey In Flowerless Neighborhoods

**DOI:** 10.1038/s41598-017-09456-y

**Published:** 2017-08-23

**Authors:** Camila Vieira, Eduardo N. Ramires, João Vasconcellos-Neto, Ronei J. Poppi, Gustavo Q. Romero

**Affiliations:** 1 Pós-graduação em Ecologia, Universidade Estadual de Campinas (UNICAMP), CP 6109, CEP 13083-970 Campinas-SP, Brazil; 2 Departamento Acadêmico de Química e Biologia, Universidade Tecnológica Federal do Paraná, CEP 80230-901 Curitiba-PR, Brazil; 3 Departamento de Biologia Animal, IB, Universidade Estadual de Campinas, CP 6109, CEP 13083-970 Campinas-SP, Brazil; 4 Instituto de Química, IQ, Universidade Estadual de Campinas, CEP 13083-970 Campinas-SP, Brazil

## Abstract

One fundamental question in prey luring systems is to understand how visual signals are interpreted by the receiver. Predators lure prey by falsely imitating the signal of a model, or may exploit sensory preferences of the receivers, which search for rewarding signals. Crab spiders reflect ultraviolet (UV) light, ambush pollinators on flowers, and manipulate flower UV signals altering the behavior and response of prey. Whereas crab spiders typically depend on flowers to forage, adult *Epicadus heterogaster* departs from this standard behavior by preying on pollinators upon green leaves, even in the absence of flowers nearby. This species has a conspicuous abdomen resembling the shape of a flower, which may reflect UV signals similar to that of flowers, and thus attract pollinators. Nevertheless, no empirical evidence is available that *E. heterogaster* foraging on leaves mimics flowers, nor how this crab spider interacts with its prey. Field and laboratory experiments demonstrated that UV reflection of adult *E. heterogaster* is the main signal responsible for the attraction of pollinators. This is the first study to demonstrate that a crab spider attracts pollinators regardless of flower UV signal, which may represent an evolutionary pathway beyond the dependence of flowers.

## Introduction

Mimicry occurs when one species, the mimic, emits a signal similar to that of a model species to deceive the receiver with the purpose of avoiding predation or capturing prey^1–3^. Ever since Bates^4^ investigated color mimicry in butterflies in the Amazon, mimicry systems have been explored either from the perspective of the mimic emitting signals to deceive the predator (protective mimicry) or the mimic emitting signals to deceive potential victims, thereby increasing the odds of well-succeed attacks (aggressive mimicry)^5–7^. In aggressive mimicry, predators or parasites lure and attract prey by falsely imitating the signal of a model^5, 8^. This is the case of assassin bugs that hunt web-building spiders by mimicking the vibrations generated by insect prey^8^, and of bolas spiders which imitate the pheromones from female moths to attract the males^8, 9^. Recently, the orchid mantis *Hymenopus coronatus* was demonstrated to mimic flowers and to attract and lure their pollinator prey at rates even higher than flowers^5, 6^.

Alternatively, the attractiveness in prey luring systems may also happen regardless of mimicry, when the predators induce a deceptive response in the sensory bias of the receiver^10–12^. For instance, some orb-web spiders, like *Gasteracantha fornicate*, have conspicuous body coloration and lure prey by inducing a deceptive signal, which is not necessarily similar to a model or object that might induce prey interest^10, 11^. Instead, it maximizes color and luminance contrast with background^10, 11^. Additionally, these polymorphic spiders and sympatric flowers exhibit notable color convergence for receivers^12^. It is likely that this predator is exploiting sensory preferences of the receiver for rewarding signal flowers^11, 12^. In cases in which deceptive signals resemble a more specific signal, polymorphism can evolve by combining multiple models^10^. Thus, the diversity of body color may converge with the diversity of floral color signals in space and time^10^.

**Figure 1:**
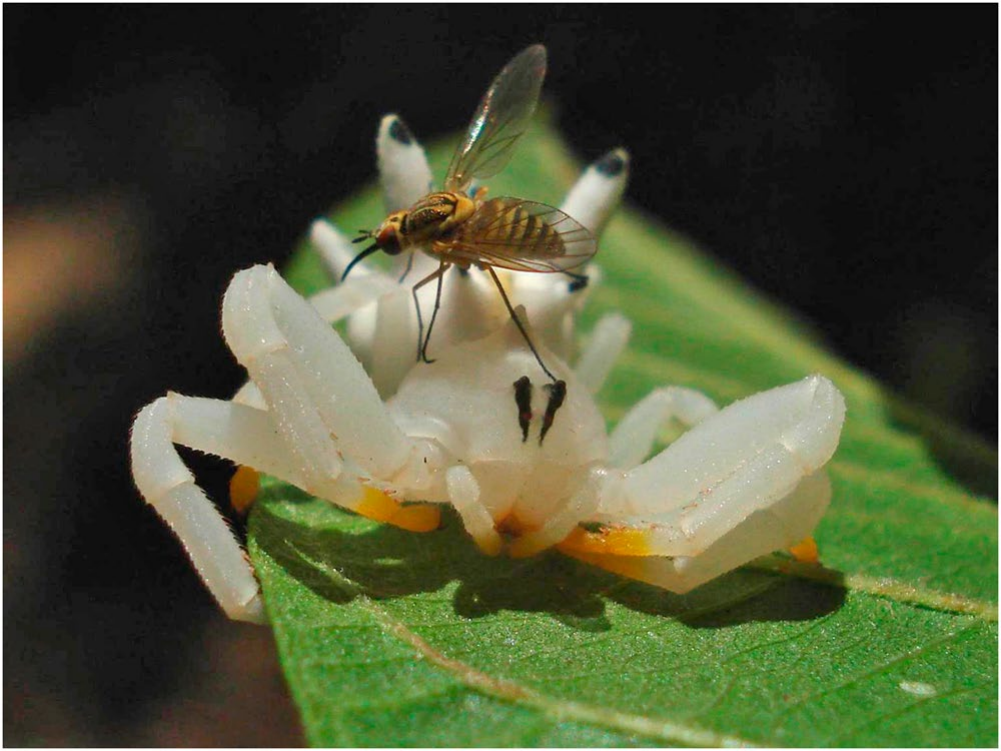
Interaction between the crab spider and a pollinator. Adult *Epicadus heterogaster* female being visited by a Xylophagidae fly upon a leaf (photo credit: Piccoli, G.C.O.).

Crab spiders (Thomisidae) reflect light in the ultraviolet (UV) wavelength range of the spectrum^13, 14^. These spiders usually ambush pollinators on flowers, are capable of manipulating flower UV signals, and thus alter the behavior and response of their prey^15, 16^. Although some crab spiders camouflage on flowers and become cryptic to their visiting prey^17, 18^, *Thomisus spectabilis* is not cryptic on flowers of *Chrysanthemum frutescens* and attracts prey by a UV color contrast of the spider against the flower petals^14^. Whereas all these spiders depend on flowers to forage, the crab spider *Epicadus heterogaster*, first observed by Bates in the Amazon, is an exception to the rule – it does not necessarily forage upon flowers. Instead, it has a conspicuous abdomen resembling a flower (Fig. 1). Unlike other crab spiders, anecdotal observations indicate that *E. heterogaster* preys on pollinators upon leaves even in the absence of nearby flowers*. Epicadus heterogaster* has abdominal protuberances that may reflect UV signals similar to that of flowers and thus attracts pollinators^19^. Capturing prey in the absence of flowers may accrue a selective advantage to crab spiders especially during seasonal periods with low availability of foraging sites. Nevertheless, no empirical evidence is yet available that *E. heterogaster* foraging on leaves can attract prey like flowers, nor how this crab spider interacts with its potential prey.

Here we manipulated UV signals to investigate whether the crab spider *E. heterogaster* is capable of attracting pollinators while on leaves. In addition, we assessed whether UV reflection in *E. heterogaster* attracts pollinators similar to floral signals. We also investigated whether crypticity of *E. heterogaster* in the pollinator visual system depends on the foraging substrate, and whether the shift in substrate from flowers to leaves as potential foraging sites depends on the ontogeny. We predict that adult spiders may reflect signals more similar to those of flowers when compared to young spiders, which are often observed foraging on flowers. Young spiders thus might be restricted to flowers as a foraging substrate, whereas adult spiders might be able to capture prey on leaves even in the absence of flowers nearby.

## Results

### Color manipulation and effect on visiting pollinators

**Figure 2:**
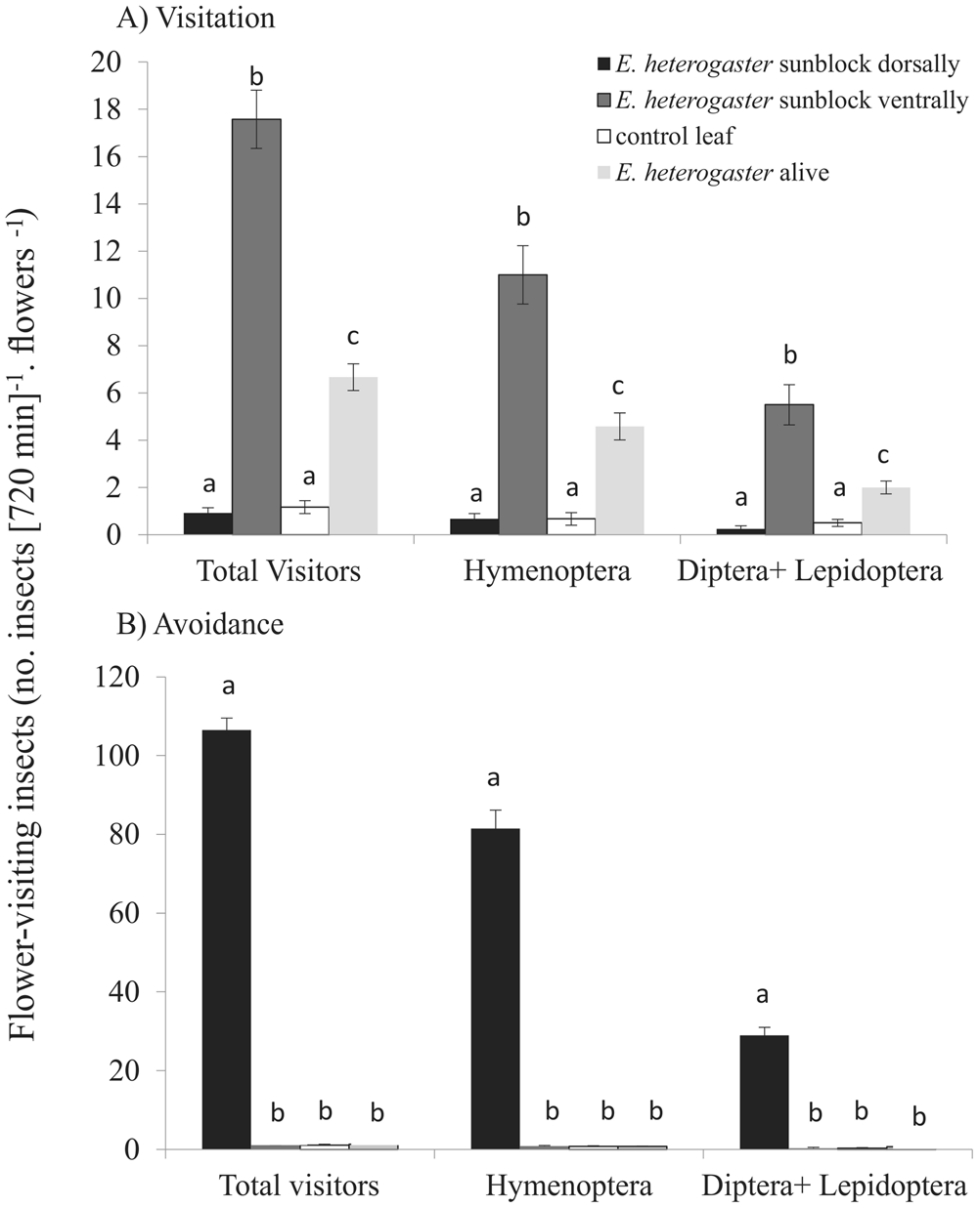
Statistical analysis of data from experiments of visitation and avoidance by pollinators. Mean number of (**A**) visits and (**B**) avoidance per 720 min of all pollinating insects (Total), as well as of Hymenoptera and Diptera+Lepidoptera on leaves. Leaves were assigned one of the following treatments: anesthetized spiders with sunscreen applied to the dorsal side, anesthetized spiders with sunscreen applied to the ventral side, unanesthetized spiders without sunscreen, and no spiders as controls. Error bars represent ± SE. Different letters indicate statistical difference (*P* < 0.05; LME/Tukey HSD, post hoc test; α = 0.05).

*Epicadus heterogaster* on leaves attracted pollinators (Fig. 2A, Table S1). Spiders with sunscreen applied ventrally (*i.e*., active UV signal) attracted several pollinators from various taxonomic groups (Table S2), whereas spiders with sunscreen applied dorsally (*i.e*., inactive UV signal) did not attract insects (Fig. 2A, Table S1). On the contrary, removal of UV reflectance repelled pollinators (Fig. 2B, Table S1). Furthermore, pollinators visited live *E. heterogaster* less than if compared to the treatments with anesthetized spiders (Fig. 2A). Insects of the order Hymenoptera were the most common visitors on leaves (Fig. 2A,B, Table S2).

### Spectral reflectance of adult and young spiders

Adult females and young spiders of *E. heterogaster* displayed high mean of UV spectral reflectance (mean percentage of reflectance between 300 to 400 nm; adult = 16.02, young = 15.32) (Fig. S1A,B). The mean spectral reflectance does not differ between adult and young spiders (Fig. S1A,B, *t* test = 0.001, df = 18, P = 1.0).

### Coloration with the hexagon color model: *Epicadus* on leaves and flowers

**Figure 3:**
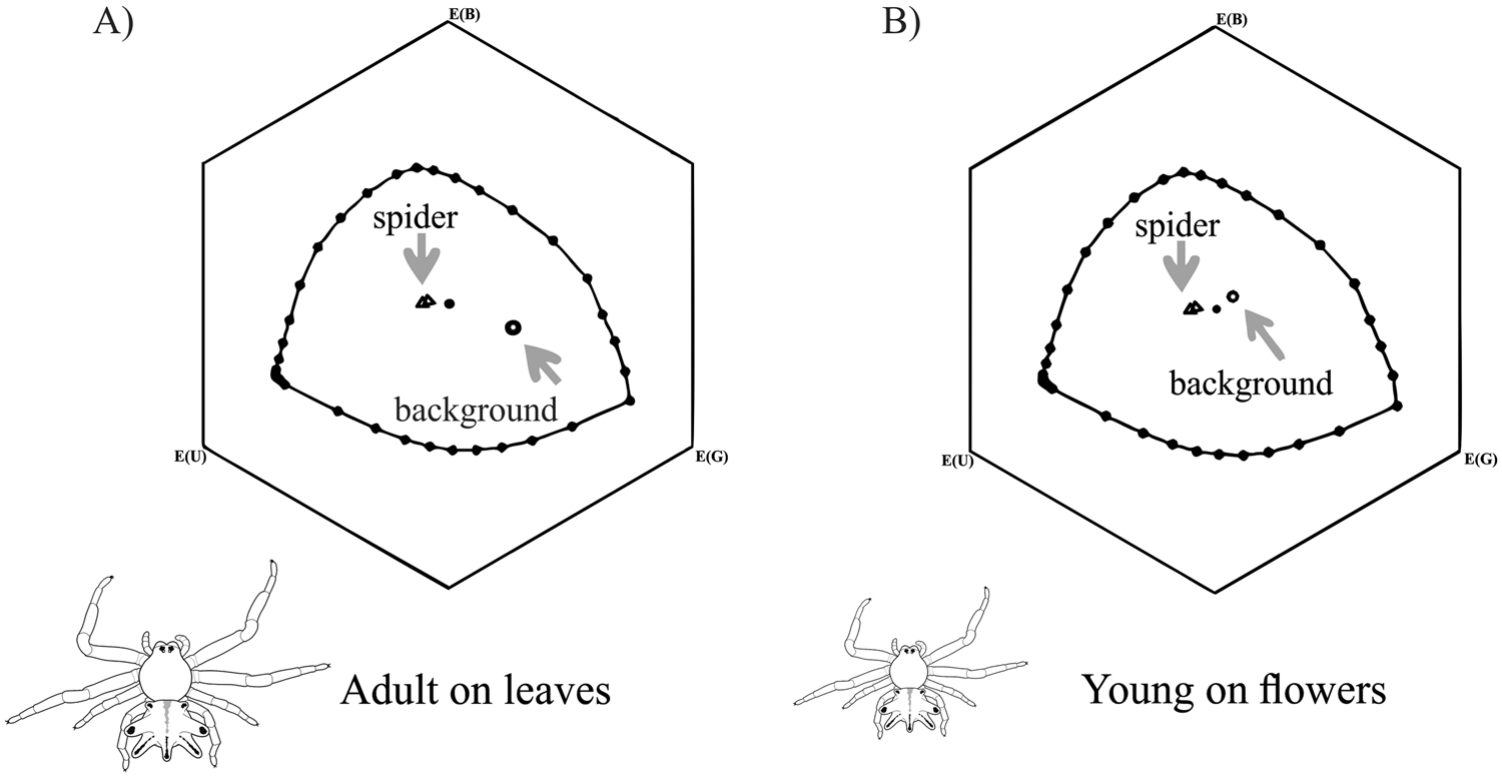
Photoreceptor stimulus representation in the visual system of bees. (**A**) Adult *Epicadus heterogaste*r on *Miconia* sp. (Melastomataceae) leaves. (**B**) Young *Epicadus heterogaster* upon flowers (credit to the spiders drawing: Soleman, R.A.).

In the visual model of Hymenoptera, bees are capable of discriminating adult *E. heterogaster* upon leaves (distance between loci = 0.15 ± 0.001; color contrast, *t* test = −30.516, df = 99, P < 0.001, Fig. 3A). However, bees cannot discriminate immature *E. heterogaster* while upon flowers (distance between loci = 0.09 ± 0.001; color contrast, *t* test = −5.137, df = 99, P = 0.97, Fig. 3B). The mean distance value of this combination is below the threshold distance of detection (0.1 hexagon units, see Methods) (Fig. 3).

### Substrate shift during ontogeny

**Figure 4:**
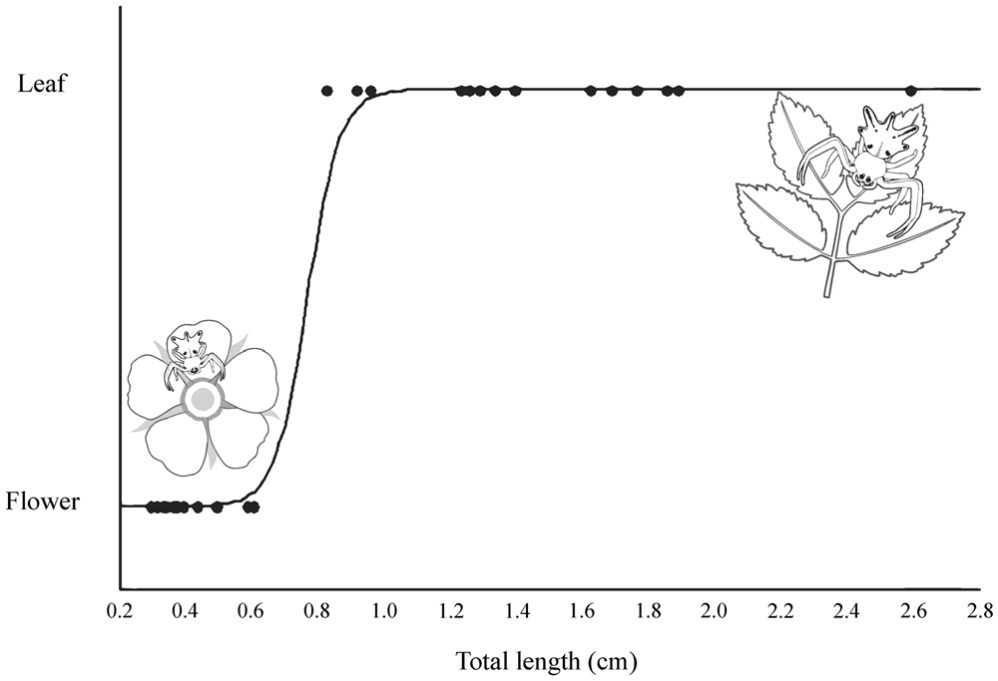
Ontogenetic habitat shift by *Epicadus heterogaster*. Logistic regression of probability of foraging upon flowers or leaves against body size in *Epicadus heterogaster* (credit to the spiders drawing: Soleman, R.A.).

*Epicadus heterogaster* with total body length between 0.29 and 0.60 cm (all young females) were observed on flowers, whereas those with total length between 0.82 and 2.59 cm (all adult females) were observed upon leaves (χ2 = 40.7, df = 1, P < 0.001, Fig. 4, Fig. S2). Based on this field survey, the shift in foraging substrate from flower to leaf should occur for a total body length in the interval between 0.60 and 0.82 cm.

## Discussion

Our results showed that adults of *Epicadus heterogaster* were able to deceive and attract pollinators regardless of substrate. The main groups of pollinators in this biological system (hymenopterans) were unable to distinguish between the spider and flower colors in a color space model. In addition, our study demonstrates empirically that UV reflection by adult *E. heterogaster* is one of the signals responsible for the attraction of pollinators. Is the ability to attract prey related to aggressive mimicry (i.e., the spiders mimic flowers), or this predator induces a deceptive response in the sensory bias of the pollinators regardless of an underlying mimicry? This question is still open for further research. But taking into account that this spider species resembles a flower, with abdominal projections similar to flower stigmas (at the human visual assessment, Fig. 1), and that it displays a pronounced color contrast with leaves, we suggest that this is a case of aggressive mimicry. In addition, differently from other flower-dwelling crab spiders, which typically forage using open forelimbs, *E. heterogaster* uses hidden forelimbs to forage (Fig. 1, Fig. S2), which makes them even more similar to flowers (i.e., they display a radial symmetry). Since crab spider forelimbs seem to be a key predator trait recognized and avoided by pollinators^16^, the behavior of hiding the forelimbs might be an additional adaptation towards flower mimicry. Under the hypothesis of floral mimicry, this case might represent an evolutionary pathway beyond the dependence of flowers in crab spiders.

UV reflectance affected pollinator visitation rate on *E. heterogaster*, i.e., when we removed the UV reflectance signals, pollinators avoided the spiders. These results indicate that UV reflectance is an important visual signal in the attraction of pollinators. Whereas previous work has shown that *T. spectabilis* (Thomisidae) reflects more UV light than the flowers on which they sit to forage, thus attracting more prey^20–22^, our study is the first one to quantitatively demonstrate prey attraction in potential floral mimicry system. Previous research has provided evidence that color contrast is more attractive to bees and other insects^17, 23, 24^, and also that there is no match between flowers and crab spider coloration^17^. Therefore, it is possible that this contrast with the substrate (leaf) is used by adult *E. heterogaster* as a strategy to attract and capture prey, and higher success could be achieved by mimicking flowers. In addition, we demonstrated here that live spiders receive less visitors than anesthetized spiders, suggesting that the movement of live spiders interfere with recognition and detection of the predator by the pollinators. Sensitivity to UV reflectance and the spider movement are hypothesized to be the factors that drive the behavior of bees visiting Australian crab spiders that forage on flowers^20^. Crab spiders with higher UV reflectance that move less are more visited by bees when compared with crab spiders that reflect less UV and move more^20^. Experiments suggest that UV reflection by crab spiders of the family Thomisidae which forage on flowers is the main attraction component of bees^13, 14, 17^. However, we suggest here that the UV signal reflected by *E. heterogaster* is pivotal for the survival of adults because they need to emit visual signals, which are sufficient and equivalent to the flower reflection signal in order to efficiently attract bees.

Our results revealed strikingly different interaction dynamics between young and adult *E. heterogaster* and their prey, concerning the use of foraging substrate (flowers or leaves). Although young and adult have similar patterns of spectral reflectance and do not differ in reflectance intensity (Supplementary Fig. S1A,B), the young are cryptic on flowers as determined by the visual model of the prey (Hymenoptera). On the other hand, adults have a highly specialized behavior and are discriminated in the visual system of bees and the green background of leaves. Since adults and young reflect UV similarly, the choice between flowers or leaves seems to depend exclusively on body size, and not on the ability to reflect UV. Young spiders, even resembling a flower, are likely too small to attract prey, i.e., the signal emitted by them is not stimulating enough to attract the receiver. Such ontogenetic shifts in foraging substrate with increasing body size are taxonomically and ecologically widespread in nature^25–30^. Nevertheless, the ontogenetic shift observed in *E. heterogaster* seems to be a first case of this phenomenon in spiders. The shift in foraging from flowers to leaves apparently takes place within a narrow interval of body size, suggesting that the switch between foraging sites may be under genetic control, as conjectured for other systems^24^. And this shift in foraging substrate could be beneficial, since far from flowers the adult spiders minimize competition and maybe predation by other predators that commonly use flowers to forage (spiders, wasps, birds). As demonstrated in this study, the exchange of visual information between *E. heterogaster* and its prey varies in the different foraging substrates and the behavioral responses of visit and avoidance may depend on the recognition of the spider against its background.

In conclusion, *E. heterogaster* reflects high UV radiation intensity and this signal seems to be responsible for attracting pollinators as a flower, regardless of the foraging substrate. However, we believe that for this lure mechanism to be successful, UV might not be the unique spider attribute, but other traits, like flower shape and specialized behaviors, may account to characterize an aggressive mimicry. Moreover, independence from flowers to attract prey by crab spiders is specific for a given stage of the ontogeny, that is, only larger ones do not depend of flower signals to capture prey. Therefore, we highlight that body shape and size are important traits in predator-prey visual systems, and should be considered in future studies.

## Material and Methods

### Study area and organism

Sampling of organisms and naturalistic observations were done in natural areas of the Atlantic Rainforest at the Biological Reserve of Serra do Japi, located near the municipality of Jundiaí, southeastern Brazil (700–1300 m). The experiments were carried out from June 2010 through April 2013. The crab spider *E. heterogaster* Guerin (1812) is rare and found on many plant species in Brazil. This spider is a sit-and-wait predator upon flowers and leaves, and can change their body color (i.e., white, yellow, purple) to match the flower color they are on or the color of nearby flowers^19, 31^. This crab spider commonly forages in shady environments where few colored items are present. Their abdomen resemble flowers and they seem to shift foraging habitats during the ontogeny. Adult females while foraging on leaves apparently mimic flowers, whereas young females apparently forage on flowers (Vieira, C. & Romero, G. Q., unpublished data).

We filmed the behavior of *E. heterogaster* for nearly 4849 hours throughout a period of three years. A protracted length of time of field observations was needed to gather data on the biology of *E. heterogaster*, particularly foraging behavior and prey capture. Sampled young individuals were kept in captivity until adults for the experiments. They were kept in plastic vials under light conditions and temperature varying from 24 °C to 34 °C and were fed every three days with insects of the families Syrphidae, Vespidae, Apidae e Hesperiidae.

### Color manipulation and effect on visiting pollinators

To investigate whether *E. heterogaster* attracts pollinators even in the absence of flowers and whether UV reflection by these spiders is responsible for the attraction of pollinators, we experimentally removed the UV reflectance by using sunscreens. We applied a mixture of two UV light-absorbing chromophores to the body of adult spiders. Both chromophores have ingredients common to sunscreens, namely, Parsol MCX (2-ethylhexyl-p-methoxycinnamate) and Parsol 1789 (4-tert-butyl-4- methoxydibenzoylmethane); the first ingredient is a UV-B light absorber, the second a UV-A light absorber (12). We carried out a randomized block field experiment in which each experimental block (n = 12 blocks) had four undamaged Melastomataceae leaves, each randomly assigned to one of the following treatments: (1) anesthetized spiders with sunscreen on the dorsal region (to block UV reflection), (2) anesthetized spiders with sunscreen on the ventral region (to control for any effects of sunscreen), (3) active and live spiders without sunscreen and, as controls, (4) leaves without spiders with sunscreen on the adaxial region. Experiments begun at 8:30 am and ended 17:30 pm. Leaves in each experimental block were filmed simultaneously in sections of 540 min each, corresponding to one day, with a Handycam Sony SD DCR-SX22, totaling 6480 min of filming. This method was chosen due to the low visitation rate of *E. heterogaster* throughout the day. The recordings were done with the same color morph (i.e., white). The number of visits and avoidances by pollinators were recorded for each section. Visiting insects were collected after the experiment for identification to the lowest taxonomic level. We defined the term “visitation” when the insect landed on the spider and stayed there for at least two seconds and “avoidance” when the insect flew towards the spider but avoided landing on the spider and turned to another nearby flower or flew away. With the exception of live spiders, exposed spiders were chilled in the freezer to prevent any influence on the behavior of the pollinator insects.

The visitation and avoidance data from the experiments were analyzed with linear mixed models (LME)^32^; blocks were treated as random effects and treatments as fixed effects. To control for lack of homogeneity when necessary, we used the varIdent function to model different variations for each level of a factor and it can be used to fit a heteroscedastic model to the data^32, 33^. To compare between treatments we used a post hoc Tukey’s test^33^. The analyses were performed on R version 3.3.2 with the function lme in package “nlme”^34^.

### Spectral reflectance of young and adult spiders

To investigate the UV reflection intensity in juvenile and adult *Epicadus heterogaster* we measured spectral reflectance between 300 and 700 nm in the abdomen of the spiders, in the petals of flowers (n = 10 young) and on the leaves of *Miconia* sp. (Melastomataceae) (n = 10 adults). We used a Cary 5000 UV- Vis- NIR (Varian, Australia) spectrophotometer with a high photometric performance in the spectral range of 175–3300 nm. We used two achromatic controls: black and white as calibration backgrounds prior to measuring each sample. We measured the spectra of spiders, leaves, and flowers 10 times and used the average number of spectra to compute the Euclidian distance in the color space model of the visual system of Hymenoptera^35, 36^. All measurements are done with the same color morph (i.e., white). We carried out the analyses in the Laboratório de UV-Vis-NR/Luminescência/Polarimetria at the Instituto de Química da Universidade Estadual de Campinas (UNICAMP).

### Coloration with the hexagon color model: *Epicadus* on leaves and flowers

We chose the Hymenoptera visual model system because although *E. heterogaster* is visited by a number of insect orders, previous experiments carried out during this study demonstrated a larger number of visits by insects of the order Hymenoptera (the authors, personal observations). In the visualization model of color space of Hymenoptera, the borders of the hexagon represent the photoreceptors and the possible combinations between them according to the spectral reflectance of the object that is being visualized. The excitation values (E) of the receptors were calculated for the photoreceptors of *Apis melliera* with their sensitivities in the ultraviolet, blue, and green peaks^36, 37^. These values range between 0 and 1. We used a background of green leaves with forest shade illumination standard^38, 39^. We also used a function of the spectral sensitivity of the photoreceptors of *A. mellifera* as reference model to understand the detection of the spider in the visual system of the Hymenoptera^35, 40^.

We calculated the mean Euclidian distance in hexagon units between the spectra of spiders, leaves, and flowers. As a reference, bees were capable of discriminating about 70% between the colors of objects and the background within distances of 0.1 hexagon units of detection^36, 41^. Therefore, we compared the distances calculated with the discriminant threshold using a *t*-test for *E. heterogaster* on flowers and leaves (color contrast). We calculated a mean Euclidian distance in hexagon units in the following combinations*: E. heterogaster* vs. flower and *E. heterogaster* vs. leaf. When the mean Euclidian distance in hexagon units between these combinations is equal or below the threshold detection distance (0.1 hexagon units) bees should not be capable of discriminating the spider from the background. When this mean value is above the threshold, bees should be capable of discriminating these spiders.

### Substrate shift during ontogeny

To test whether a shift in substrate between flower and leaf in *E. heterogaster* depends on the ontogeny, we fitted a logistic regression modeling the probability of occupation of a given substrate as a function of the total length of the spider. Model parameters were estimated by maximum likelihood and the significance was assessed by the Likelihood Ratio Test (LRT;  ${{\rm{\chi }}}^{2}$ statistic) with one degree of freedom and a prescribed P value. We used data from 30 female *E. heterogaster* collected along trails in the Serra do Japi and fitted these data with a logistic regression using the Logit binomial model in R version 3.3.2 (R Development Core Team 2016)^34^.

## Electronic supplementary material

Supplementary Information

Movie 1

Movie 2

Movie 3

Movie 4
