# Supplementary material for: Crab Spider Lures Prey In Flowerless Neighborhoods
Source: Sci Rep. 2017 Aug 23;7:9188. doi: 10.1038/s41598-017-09456-y (PMC5569008; doi:10.1038/s41598-017-09456-y)
Supplement: Supplementary file 1 — Supplementary Information [file 41598_2017_9456_MOESM1_ESM.pdf]

Supplementary Information

Crab spider lures prey in flowerless neighborhoods

Camila Vieira, Eduardo N. Ramires, João Vasconcellos-Neto, Ronei J. Poppi & Gustavo Q. Romero*

*Corresponding author. E-mail: gqromero@unicamp.br

This file includes:
Figs. S1 and S2
Table S1 and S2

Other Supplementary Information for this manuscript include:

Movie S1. Active and live spiders without sunscreen preying hoverflies
Movie S2. Active and live spiders without sunscreen trying to prey hoverflies
Movie S3. Active and live spiders without sunscreen trying to prey butterfly
Movie S4. Anesthetized spiders with sunscreen on the ventral region

Supplementary Figures

Supplementary Figure S1. Statistics of reflectance for *Epicadus heterogaster*. (A) Mean percentage of reflectance curves ±SD of *Epicadus heterogaster* adult abdomens. (B) Abdomens of young *Epicadus heterogaster*. Spectral reflectance was measured from 300 to 700 nm. The left side of the dashed line indicates the UV wavelength. Credit to the spiders drawing: Raduan A. Soleman.

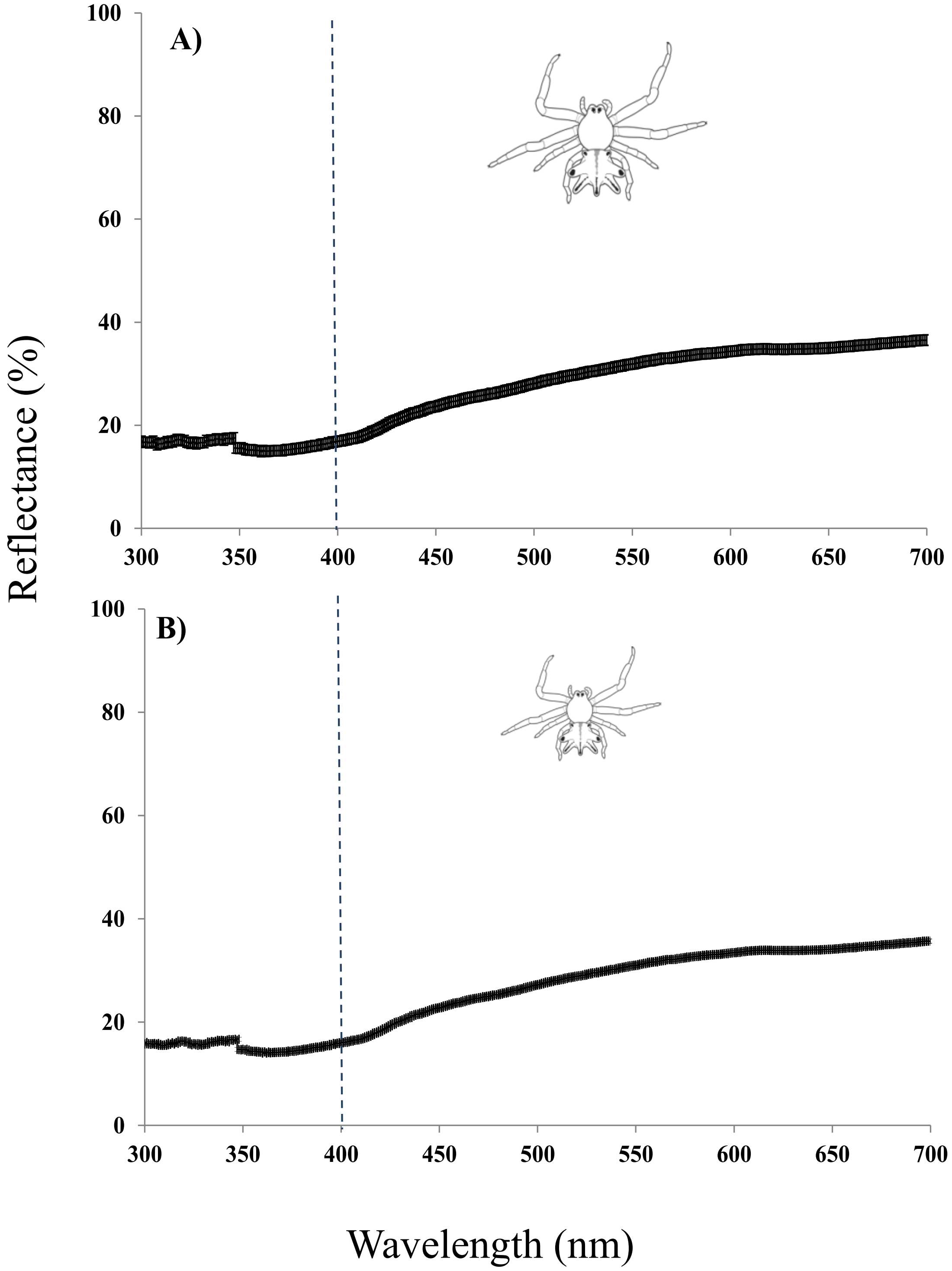

Supplementary Figure S2. *Epicadus heterogaster* A) adult upon a leaf, **(**B) young on a flower. Photo credits: A) Andressa Degressi, B) Tiago Nascimento Bernabé.

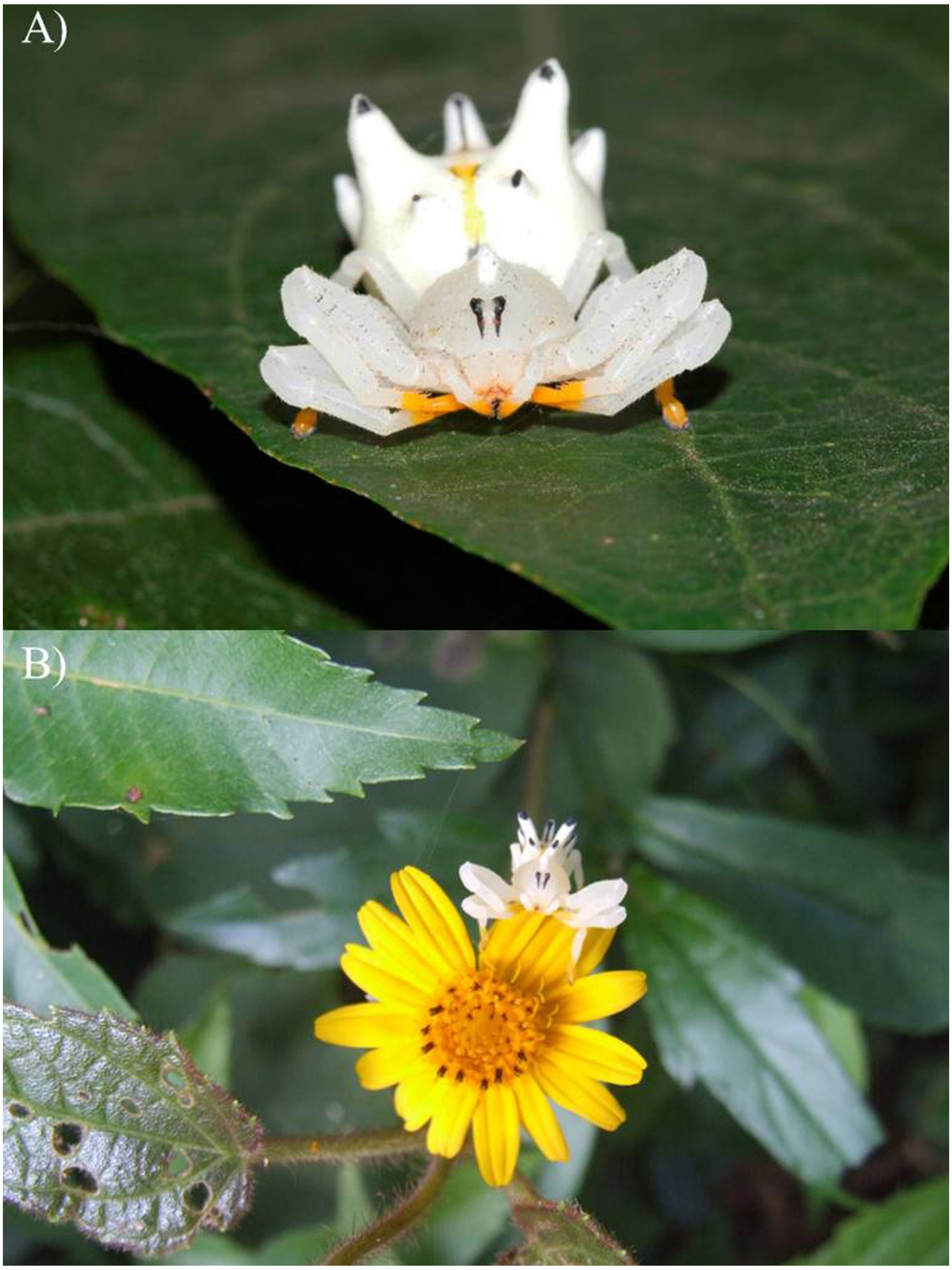

Supplementary Tables

Supplementary Table S1. **Linear mixed effects models (LME) to assess the significance of the fixed effects (*i.e.,* treatments)**. The treatments were: leaves with *Epicadus heterogaster* with sunscreen applied dorsally, leaves with *Epicadus heterogaster* with sunscreen applied ventrally, leaves without crab spiders and leaves with live *Epicadus heterogaster* in the visiting behavior and total avoidance of pollinators, as well as hymenopterans and lepidopterans (Degrees of freedom for the numerator =3, Degrees of freedom for the denominator =34).

**Variation among treatments**	*F*	*P*
**Total visitation**	85.768	P<0.001
**Visitation by Hymenoptera**	50.835	P<0.001
**Visitation by Lepidoptera+Diptera**	22.460	P<0.001
**Total avoidance**	405.641	P<0.001
**Avoidance by Hymenoptera**	101.219	P<0.001
**Avoidance by Lepdoptera+Diptera**	197.923	P<0.001

Supplementary Table S2. List of floral visitors. The visitors were observed visiting *Epicadus heterogaster* upon leaves during the experiment.

**Order**	**Family**	**Morphospecies**
Diptera	Asilidae	Asilidae sp.1
Diptera	Asilidae	Asilidae sp.2
Diptera	Syrphidae	Syrphidae sp.1
Diptera	Syrphidae	Syrphidae sp.2
Diptera	Syrphidae	Syrphidae sp.3
Diptera	Xylophagidae	Xylophagidae sp.1
Hymenoptera	Apidae	*Apis melífera*
Hymenoptera	Apidae	Apidae sp.1
Hymenoptera	Apidae	Apidae sp.2
Hymenoptera	Apidae	Apidae sp.3
Hymenoptera	Apidae	*Bombus brasilienses*
Hymenoptera	Apidae	*Bombus* sp.
Hymenoptera	Apidae	*Melipona* sp.
Hymenoptera	Apidae	*Trigona* sp.1

Hymenoptera	Apidae	Trigona sp.2
Hymenoptera	Vespidae	*Agelaia vicina*
Hymenoptera	Vespidae	*Agelaia* sp.
Hymenoptera	Vespidae	*Omicron tuberculatum*
Hymenoptera	Vespidae	*Polybia* sp.1
Hymenoptera	Vespidae	*Polybia* sp.2
Hymenoptera	Vespidae	*Polistes versicolor*
Hymenoptera	Vespidae	*Polybia fastidiosus*
Hymenoptera	Vespidae	*Pachymenes* sp
Hymenoptera	Halictidae	Halictidae sp.1
Hymenoptera	Halictidae	Halictidae sp.2
Lepidoptera	Hesperiidae	Hesperiidae sp.1
Lepidoptera	Hesperiidae	Hesperiidae sp.2
Lepidoptera	Hesperiidae	Hesperiidae sp.3
Lepidoptera	Hesperiidae	Hesperiidae sp.4
Lepidoptera	Hesperiidae	*Urbanus chalco*
Lepidoptera	Nymphalidae	*Aeria olena*

Lepidoptera	Nymphalidae	Nymphalidae sp.1
Lepidoptera	Nymphalidae	*Phyciodes claudina*
Lepidoptera	Pieridae	*Appias drussila*
Lepidoptera	Pieridae	Pieridae sp.1
